# The persisting presence of absence in female sex development: a critical interdisciplinary reflection

**DOI:** 10.1186/s13293-026-00848-2

**Published:** 2026-03-25

**Authors:** Birgit Stammberger, Xenia Steinbach, Nadine Hornig

**Affiliations:** 1 Institute for History of Medicine and Science Studies (IMGWF), University of Luebeck, Luebeck, Germany; 2 Institute for Ethics, History and Philosophy of Medicine, Hannover Medical School (MHH), Hannover, Germany; 3 Institute of Human Genetics, University Hospital Schleswig-Holstein and Kiel University, Kiel, Germany

**Keywords:** Absence-theory, Female sex development, Oestrogen action, Knowledge production, Interdisciplinarity

## Abstract

**Background:**

For much of the 20th-century, developmental endocrinology was structured around a binary model that positions male differentiation as an active, hormone-driven process and female development as the passive consequence of androgen absence. This framework has profoundly shaped both experimental practice and conceptual understanding in reproductive and developmental biology. Yet, empirical evidence in molecular endocrinology, combined with insights from feminist science studies and the history of science, invites a revitalization of the long-standing critique of the persistence of this model.

**Main body:**

This review critically re-interrogates the longstanding notion of female sex development as an outcome of mere androgen deprivation. First, through a historical analysis of key experimental systems in 20th-century embryological endocrinology, we trace how this conceptual pattern emerged and became stabilized within the discipline. We show how the methodological privileging of androgenic mechanisms over other hormonal pathways contributed to defining femaleness as absence. Second, drawing on research in developmental and molecular endocrinology, we review the roles of oestrogens and their receptors in mammalian female genital development. Synthesizing these findings, we support a less reductionist model that opens the possibility to more research on oestrogen-dependent female sex differentiation and defines female sex development as an active, regulated process rather than a default state. Finally, we situate the 'absence' model of femaleness within its broader cultural and symbolic contexts. Through a material-semiotic analysis, we demonstrate how scientific concepts of sex are co-constituted with wider social meanings, and how this interplay shapes what is rendered visible or invisible in biological research.

**Conclusion:**

Emerging from a multidisciplinary dialogue between biomedicine, the history of science, and feminist science studies, our review highlights how cultural assumptions of gender are embedded within scientific practices of analyzing sex-differences. By integrating reflexive humanities perspectives with empirical biomedical research, we argue for a more accurate and equitable understanding of female development - one that recognizes oestrogenic activity as central to sex differentiation and challenges the reduction of femaleness to hormonal absence. This cross-disciplinary engagement illustrates the transformative potential of re-examining foundational scientific paradigms through collaborative, critical inquiry.

**Plain summary:**

Research on how sex develops in mammals was based for a long time on a simple binary model: male development is an active process driven by androgens, while female development happens passively when these hormones are absent. Our article re-challenges this long-standing view by referring to the history of the concept of female sex development as a passive process and reinforcing the critical works already available on its continued persistence. First, we trace how this 'female as absence' model emerged in 20th-century developmental endocrinology. Second, we review empirical evidence showing that oestrogens and their receptors play active roles in shaping female genital development, and we present a model of oestrogen-dependent pathways in this process. Third, we situate the idea of femaleness as absence within its wider cultural and symbolic background, showing how scientific concepts are influenced by historical and social meanings. Bringing together perspectives from biomedicine, the history of science, and feminist science studies, we use a multidisciplinary dialogue to show how gender bias becomes embedded in both research design and clinical interpretation. Recognizing these biases is not only a matter of scientific precision but also of improving health outcomes - for example, in the diagnosis and care of people with differences of sex development or in the advancement of women’s health.

**Graphical Abstract:**

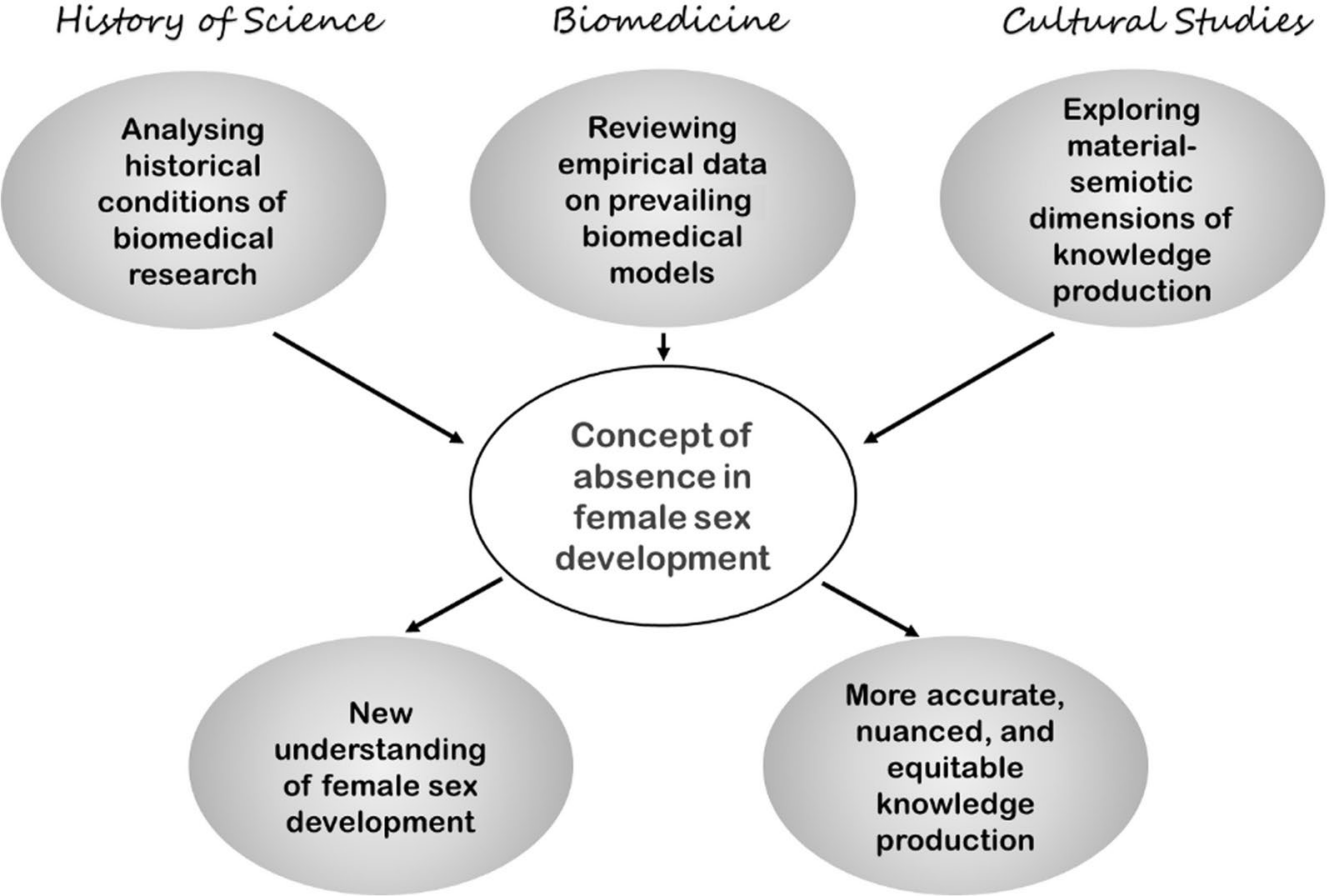

**Supplementary Information:**

The online version contains supplementary material available at 10.1186/s13293-026-00848-2.

## Highlights

Historical analysis of the 'absence-theory' of female sex development.

Marginalization of female sex differentiation in early experimental and conceptual models.

Empirical evidence for oestrogen action in female sex development.

Broader dimension and cultural meaning of femaleness as absence.

Interdisciplinary collaboration between history of science, biomedicine and feminist science studies.

**Supplementary Information:**

The online version contains supplementary material available at 10.1186/s13293-026-00848-2.

## Background

Biological research on sex development uses experimental models to identify sex-specific factors that guide these processes. These models are not merely objective reflections of natural phenomena; they are also shaped by the epistemological, conceptual, and cultural assumptions within which knowledge about sex development is constructed. A commonly dominant model is based on a binary logic of presence versus absence, in which male sex development mechanism is associated with active biological intervention, whereas the female sex development is framed as a passive or default mechanism.

This framing is deeply embedded in the history of genetics and endocrinology. Female development has often been described in terms of what is missing - whether hormones, a Y-chromosome, or a specific genetic trigger - while male development is portrayed as the result of active processes. The active/passive model has its roots in the discovery of androgens as the principal agents promoting the differentiation of the male genitalia, while their absence was considered to be sufficient for female sex differentiation. The discovery of the *SRY* gene in 1990, understood as the molecular switch initiating testis formation, reinforced this narrative by positioning male differentiation as a purposeful activation, and female development as occurring in its mere absence.

However, such interpretations are neither neutral nor objective. Instead, they reflect and reinforce the gender-related assumptions embedded in the epistemological frameworks through which scientific knowledge is produced. In this context, gender is understood as a social and cultural category, encompassing the norms, relationships, roles, and expectations that are assigned to individuals, whereas sex refers to the biological characteristics, such as chromosomes and hormones [1]. Drawing on the concept of ‘gender in science’ [2], we adopt a feminist epistemological approach to examine this androcentric bias [3], arguing that sex and gender are not entirely separate, but rather interact continuously.

Since the 1970 s, several scientists in the academic field of biology have critically reflected androcentric bias in sex development research [4, 5]. Later feminist science studies questioned the gender biases inherent in scientific inquiry [6–8]. Building on this critique, feminist researchers have developed new approaches and tools to identify and overcome the androcentric bias in biological research on sex development [9–11]. Feminist scholars like Anne Fausto-Sterling, particularly in her book “Sexing the Body” [12, 13], critiqued the concept of the female as a lack or an absence, arguing that it is rooted in gendered cultural metaphors rather than mere biology [9, 14, 15]. Scientific findings also began to show that female development requires active signalling processes, involving genes like *WNT4*, *RSPO1*, and *FOXL2*, challenging the model of passive female sex development [16–18]. Regarding hormone-dependent sex differentiation, the view prevails that androgens are actively shaping the male genitalia while the absence of androgens is sufficient to produce the female genital tract. This view persists in contemporary medical reference resources, accessible through the NCBI Bookshelf as well as in currently used textbooks for medical students and health care professionals (suppl. Table 1).

In this review, we re-question the established model of describing female sex development as occurring through the mere absence of androgens by: (1) tracing the historical emergence of this conceptual pattern through a close reading of experimental systems in 20th-century endocrinological embryology; (2) reviewing the scientific literature on the roles of oestrogens and their receptors in mammalian female sex development and providing an updated model of oestrogen dependent female genital development; and (3) situating the concept of femaleness as absence within broader cultural and symbolic contexts, highlighting the material-semiotic dimensions through which scientific knowledge is produced and stabilized.

This article emerges from an interdisciplinary dialogue between molecular bioly, history of science, and feminist science studies, using this exchange as a lens to examine how femaleness has been associated with biological absence. We show how dominant scientific models are shaped by broader social narratives that continue to influence biomedical knowledge. By foregrounding the dialogue between biomedicine and the humanities, we aim to bridge the gap of the ‘two cultures’ of sex and gender [19, 20] and demonstrate the potential of reflexive collaboration in rethinking scientific knowledge production.

## Historical perspective on the ‘absence-theory’ of female sex development and analysis of 20th-century experimental models

### Early formulations of the 'absence hypothesis'

The first explicit formulation of the hypothesis of absence in connection with female sex development dates back to the 1930s. The Austrian physiologist Berthold Paul Wiesner conducted experimental studies on newborn rats. He treated female newborn rats with the synthetic androgen proviron for two weeks and observed a virilizing effect with increased clitoral growth. When castrating male newborn rats, he noticed a clitoral development comparable to female rats. Based on the latter, he described female sexual differentiation “as the absence of any, rather than the presence of a specific sex hormone” and referred to this as the “absence-presence theory” [21]. He further specifies that “absence of this extracellular factor (male hormone) permits intracellular factors to become effective” concluding that non-hormonal factors can lead to female development if not repressed by androgens [21]. Wiesner thus took part in an international scientific controversy that had been going on at that time as to whether the hormones of the testicles and ovaries were each involved in embryonic sex development (‘dihormonic theory‘) or whether only the male gonadal hormones played a differentiating role (‘monohormonic theory‘) [13].

### Endocrinological debates and the indifferent embryo

This debate arose because, on the one hand, in the 1910 s, endocrinological experiments on postnatal mammals had already clearly shown that both the active substances of the testicles and the ovaries had sex-specific effects, suggesting a dihormonic theory of embryonic sex differentiation. Above all, it was the sex change experiments of Austrian zoologist Eugen Steinach, who gained international fame by transplanting the gonads of male and female guinea pigs and “arbitrarily changing” their biological sex or creating “artificial hermaphrodites” [22, 23].

On the other hand, early endocrinological research on embryonic sex development left the role of the ovaries and oestrogens unclear. This research ties in with the idea, dating back into the early 19th-century, of an ‘indifferent sexual disposition’ of embryos, and thus to the assumption that embryos in the early stages of their development did not yet have a morphological sex and only developed this in the course of sex-specific embryonic development processes [8, 24]. It raised the question of the physiological factors that mediated this very development.

At the end of the 19th-century, long-standing cultural notions that located the essence of femininity and masculinity in the sexual organs became intertwined with the emerging science of endocrinology [25, 26]. This convergence gave rise to the hypothesis that certain active substances produced by the fetal gonads might govern subsequent processes of sex differentiation. Although these substances could not yet be chemically identified, they were already being discussed as potential mediators of biological sex development [27]. First histological-morphological studies on gonads of pig embryos showed an early differentiation of hormone gland cells in the testicular tissue of the embryos and an optical enlargement of these cells at the time of their further sexual differentiation, which was interpreted as evidence of the active involvement of the testicular glandular secretions in this differentiation process [28]. However, no comparable differentiation of glandular cells was found in female embryos. While these investigations thus established a first tangible link between the male gonadal agents and male embryonic sex development, the mechanisms of female sex development remained unknown and appeared to be absent under the microscope. Furthermore, studies on freemartins - chromosomally female animals (usually domestic cattle) from twin or multiple births that are born virilized and remain sterile - reinforced the relevance of male gonadal hormones in fetal sex development and once again left the mechanisms of female sex development largely unexplained. Their anatomical analyses showed that the mixed-sex embryos connected via the placenta sometimes formed anastomoses, i.e. common blood vessels. This led to the conclusion that the masculinizing effect could result from the transfer of male hormonal substances from the male twins into the female fetus [29, 30]. Why these substances were dominant over the female differentiation process remained an open question, as did the chemical nature of the male hormones.

### First experiments on fetal hormone action

The elucidation of the chemical structure and the pharmaceutical industrial production of androgens and oestrogens in the 1930s made new experimental models possible in which the gonadal hormones could be actively applied in embryos. However, even here, only androgens showed clear sex-specific effects in experiments on mammals, while oestrogens produced partly contradictory, sometimes highly destructive and even lethal effects in guinea pigs of both sexes [31]. Still, none of these models clearly identified the ovaries and oestrogens as irrelevant for fetal sex differentiation [31]. Although the testis was considered pivotal in producing sex differences, it was also maintained that the ovary likely contributed to early sex differentiation by stimulating Müllerian structures and possibly inhibiting Wolffian ducts, indicating complementary roles of both gonads [32].

The question of the mechanisms underlying female sexual differentiation in mammals receded into the background when the French embryologist Alfred Jost succeeded in repeating Wiesner’s castration experiments on fetuses - in his case rabbits and not rats - at the end of the 1940s. His experiments substantially shaped the understanding of hormone dependent mammalian sex differentiation until today.

## Reassessing the passive model of female reproductive tract development

### Jost’s foundational experiments and the male-active paradigm

**Fig. 1:**
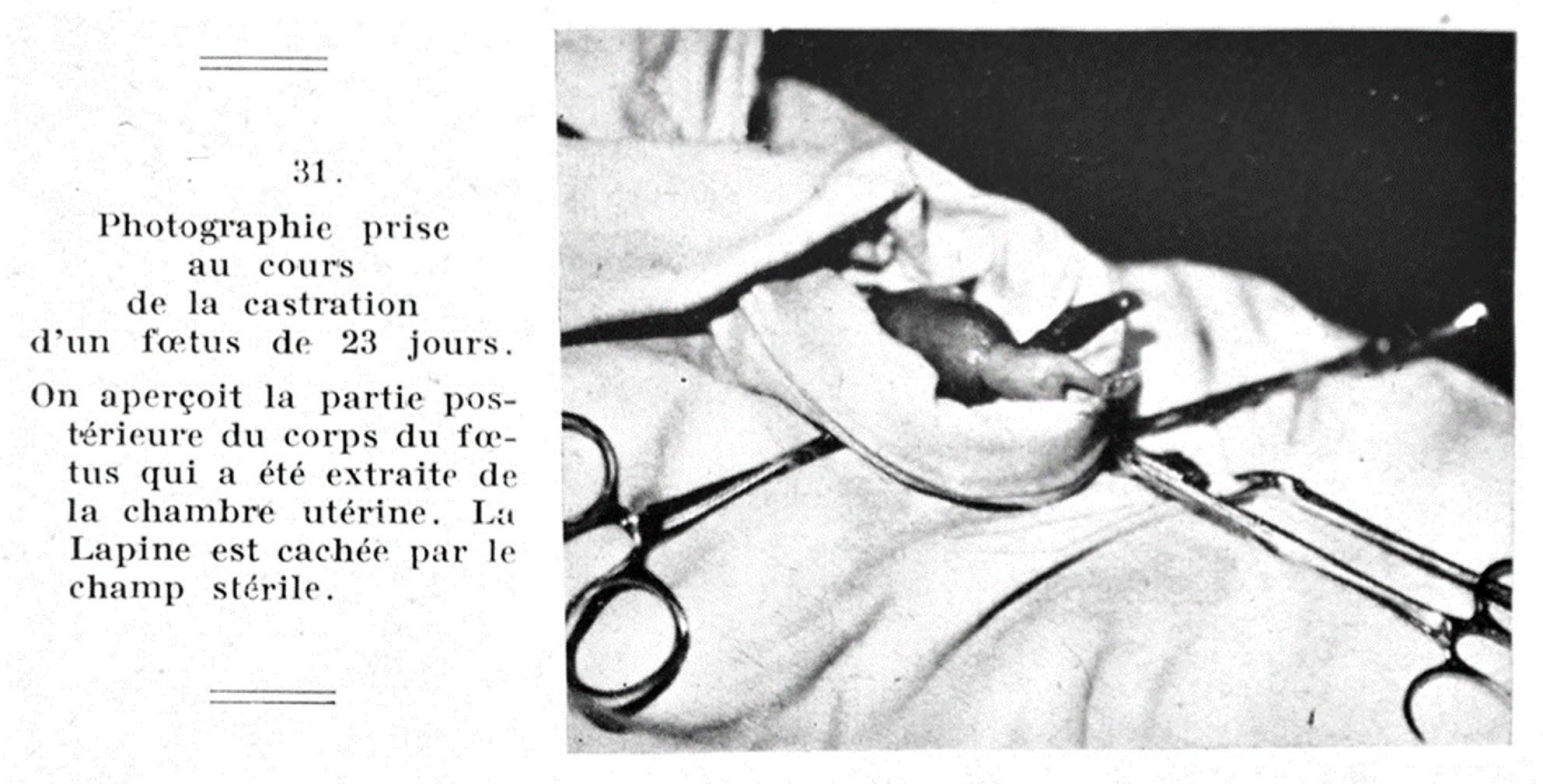
Castration procedure on a 23-day old rabbit fetus [32]: 275)

**Fig. 2:**
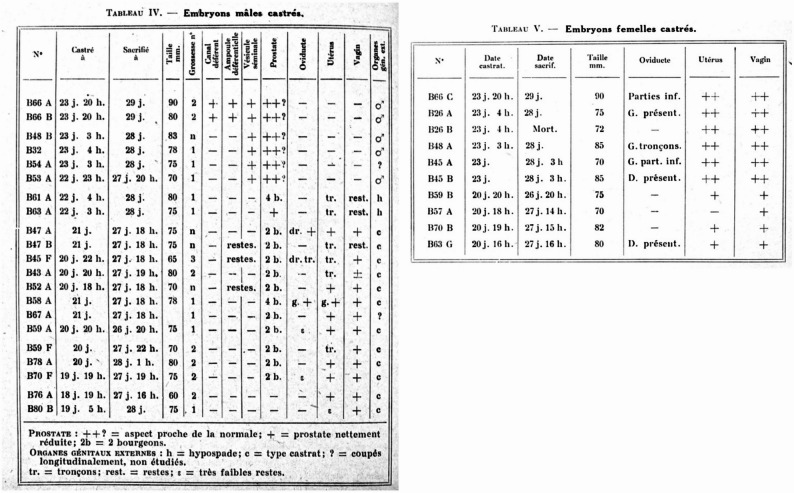
Phenotypic description of castrated animals. Left panel [32]: 278), right panel [32]: 286)

In his foundational experiments Jost removed rabbit gonads at different timepoints of fetal development, let the fetuses develop to term and analyzed their genital system immediately after birth (Fig. 1). He observed that testicular ablation led to some degree of differentiation of the Müllerian ducts - ducts out of which the fallopian tubes, the uterus, and the upper vagina develop. He also observed little to no development of the Wolffian duct (which normally forms the male internal genitalia) and little to no development of the male external genitalia, depending on when the castration occurred (Fig. 2). By inserting testosterone crystals during castration, Jost could restore the male phenotype, concluding that testosterone is necessary for male genital development. As testicular Sertoli cells produce anti-Müllerian Hormone (AMH) during male fetal development, the Müllerian structures were preserved [32].

Less known is that Jost also performed castrations on female rabbit fetuses. Although the number of operated female fetuses was smaller and the earliest operation occurred nearly two days later than in males, Jost observed that removal of fetal ovaries resulted in hypoplastic or absent uteri and underdeveloped vaginas (Fig. 2). This opened up the assumption, that also the development of the female genitalia may be an active process. However, Jost´s experiments on female fetuses remained incomplete, as he did not perform very early ovariectomies - the most significant effects on male differentiation were observed with earlier interventions - and as he did not perform oestrogen replacement during female castrations to revert the phenotype. Nevertheless, Jost mentioned that hormonal secretion from the fetal ovary could not be excluded [32].

More than 20 years later, Jost’s observations of effects on female reproductive tract development as a hormone-independent process became a “matter of fact” [33]. He emphasized that “several observations indicate that both sexes are not equal or equipotential as to their developmental trends and mechanisms” as “testes rapidly differentiate whereas ovaries are first characterized mainly by the fact that they do not become testes” [33]. He further stated that the “ovary is unnecessary for the feminine differentiation” and that “in males, femaleness has to be repressed, and maleness imposed by the testes.” Considering the state of research at the time, he concluded “that throughout sexual differentiation in mammals, maleness has to be actively imposed on a system which will become feminine if it escapes control” [33]. These conclusions have shaped the understanding of sex development for decades and, continue dominating textbooks and scientific literature (suppl. Table 1). [34, 35]

### Early evidence for active female hormonal signalling

The continuation of references to the absence-presence model of female sex differentiation is surprising since several studies have challenged the notion of female development as a hormonally neutral, passive pathway. Already in the 1960 s, research on birds has shown estrone and oestradiol secretion by fetal bird ovaries [36] and in 1977, oestradiol production from radiolabeled testosterone was demonstrated in rabbit fetal ovaries [37]. The authors observed that the onset of the conversion of testosterone to oestradiol in the ovary coincided with the initiation of testosterone synthesis in the fetal testis (day 18), suggesting that both processes may be regulated by similar developmental cues. Importantly, Jost’s earliest female castration was performed at 20 days and 16 h – nearly two days later than in males (18 days and 19 h). Thus, oestrogen may already have been present in the female fetus at the time of castration, potentially accounting for the comparatively weaker effects observed following ovarian removal in females.

Two years later similar results were obtained in human fetal gonads. The conversion of radiolabeled androgen to oestrone (E1) and 17ß-oestradiol (E2) was assessed in tissues of human fetuses from phenotypically indifferent stages to mid-gestation. Oestrogen synthesis was undetectable in testes, but present in fetal ovaries from around 8 weeks post-conception (wpc), prior to histological differentiation [38]. A more recent study confirmed the detection of increasing E1 and E2 levels in the first and second trimester in fetal ovarian tissue [39]. Gene expression data show that oestrogen receptor 1 (ESR1) expression in human fetal ovaries rises from 8 wpc and peaks at 16 wpc, while oestrogen receptor 2 (ESR2) rises from 6 wpc and peaks at 11 wpc [40]. Therefore, both ligands and receptors are present in the ovary at early stages of human ovarian development. In reptiles and birds, ovary development is actively driven by oestrogen. Traces of this pathway remain in marsupials, close relatives of eutherian mammals [41]. Although sex determination in humans is genetically controlled, early ovarian expression of oestrogens and their receptors suggests a possible role for oestrogen signaling in this process. In line with this, oestrogens have been shown to increase ovarian granulosa cell markers FOXL2 and WNT4 and decrease testicular Sertoli cell markers SRY, SOX9 and AMH in the human testicular cancer derived embryonic cancer cell line NT2/D1 [42]. These findings support the idea that endogenous ovarian oestrogens may contribute to the patterning and growth of female internal genitalia. They suggest that the developmental trajectory of the female reproductive tract is a hormonally orchestrated process dependent on the presence of oestrogens - and not simply a passive default.

### Rethinking placental oestrogens in fetal sexual differentiation

A central question is whether maternal and placental oestrogens could drive female genital development - and how the male fetus avoids feminization. The default hypothesis is in line with a “passive” exposure of the female fetus to placental oestrogens, whereas the male fetus must actively counter high placental oestrogen levels by producing testosterone during the critical developmental window. Although influential, this view has notable limitations.

First, placental oestrogens primarily regulate maternal physiology - supporting vasodilation, uteroplacental blood flow, angiogenesis, and metabolic adaptation to ensure fetal growth [43, 44]. Amniotic fluid instead is a useful source to measure fetal hormone production as it contains mainly fetal secretions. Studies show higher testosterone levels in amniotic fluid from male than from female fetuses between 14 and 20 weeks of gestation, indicating fetal testicular activity [45, 46], and some report higher estradiol levels in female fetuses [38, 39, 45], although others find no sex-specific oestrogen differences [46].

Second, maternal oestrogen concentrations rise steadily throughout pregnancy [47, 48]. The highly ordered, tissue-specific events of sexual differentiation cannot be explained by passive exposure to these steadily rising hormones. Instead, they require precisely timed, localized steroid signaling governed by differential hormone receptor expression, and regional sensitivity [49].

Third, androgen and oestrogen bioavailability is tightly regulated. The cyto- and syncytiotrophoblast create a physical and metabolic barrier to maternal steroid transfer, and most circulating steroids are protein-bound, limiting free diffusion [50]. In humans, androgens and oestrogens are bound to the sex hormone binding globulin (SHBG), a glycoprotein produced by the liver regulating the availability of steroid hormones in the blood and their accessibility to target tissues and cells [51]. In rodents, this protein is known as the androgen binding protein (ABP). SHBG/ABP-bound steroids can enter target cells through binding to endocytotic receptors expressed in steroid-responsive tissues, like the male and female reproductive organs [52].

Importantly, placental oestrogen production in humans depends on the production of the precursor metabolite DHEAS from the fetal adrenal after the first trimester which is converted into androgens and oestrogens by the placenta [48, 53]. In rodents, maternal ovarian and placental cooperation is required throughout gestation to sustain oestrogen production [53]. Rodents additionally use α-fetoprotein, which binds oestrogens and protects the developing female brain from “defeminization” [54].

Together, these findings indicate that fetal sex development relies on tightly regulated, intrinsic, precisely timed endocrine cues - not simply on generalized exposure to placental oestrogens.

### Experimental models demonstrating oestrogen’s role

Murine knockout models have provided further compelling evidence that oestrogen signalling plays an active role in reproductive tract patterning. Female and male mice lacking oestrogen receptor α (ERα knockout; ERαKO) and ERα/ERβ female double knockouts (αβERKO) are infertile [55, 56], while female oestrogen receptor β knockout mice (ERβKO) are subfertile [56]. Interestingly, in the double knockouts, at the onset of puberty, some ovarian follicles underwent sexual trans-differentiation with ovarian granulosa cells developing into testicular Sertoli-like cells. In terms of uterine development, female mice lacking oestrogen receptor α (ERαKO) show hypoplastic, estradiol-insensitive uteri [56], and ERαKO rats develop thread-like uteri [57]. ERβ knockout (ERβKO) female mice show either no overt phenotype or immature uteri. Double knockouts exhibit severe uterine hypoplasia [56]. Female mice lacking aromatase - the enzyme that converts androgens to oestrogens - also display hypoplastic uteri [58]. Regarding the external genitalia, αERKO female mice exhibit enlarged clitorises containing cartilage, typical of the penis but never observed in the clitoris. Importantly, serum testosterone levels in these females were not elevated, suggesting that masculinization was not androgen-driven [59] and that oestrogen action through ERα is needed to prevent virilization of the external genitalia.

Further evidence for active oestrogen signalling comes from studies of *in utero* diethylstilbestrol (DES) exposure. DES is a synthetic nonsteroidal oestrogen that can bind and activate both oestrogen receptors [60]. It was historically prescribed to pregnant women to prevent miscarriage but has shown to cause developmental malformations in exposed female and male offspring. These include anomalies of the cervix and vagina in the female offspring when DES exposure occurred between 7 and 15 weeks of gestation [61]. This is the time the ovaries develop. An increased risk of hypospadias has been observed in male mice prenatally treated with DES supporting the idea that oestrogen-dependent processes can interfere with regular male genital development [62]. In female mice, DES has also been shown to disrupt the expression of *Hox* genes critical for uterine regionalization, resulting in long-term reproductive defects. These effects are absent in ERαKO mice, confirming that oestrogen acts through classical receptor-mediated pathways to program uterine development [63].

### Clinical evidence in humans

Postnatal human data also support oestrogens as inductive organizers of Müllerian structures. A systematic review reported that in 22 of 25 individuals with primary hypogonadism (a condition of compromised gonadal function) the diagnosis of absent uterus was made, including 14/14 females with a 46,XX karyotype and 8/11 with a 45,X karyotype (Turner syndrome). Uterine growth was observed upon exposure to exogenous oestrogen, leading the authors to conclude that oestrogen deficiency may explain reports of Müllerian agenesis in these conditions [64].

The ideal human model for proving oestrogen’s role in female genital development would be complete oestrogen resistance. Analogous to complete androgen insensitivity - where 46,XY individuals lack Wolffian duct and male external genitalia development due to inactivating mutations in the androgen receptor gene - complete oestrogen insensitivity in 46,XX individuals would allow direct assessment of oestrogen’s role in female sex development. So far, four homozygous missense mutations in *ESR1* have been identified in 46,XX individuals diagnosed with Estrogen Insensitivity Syndrome (EIS). Common features include absent breast development, primary amenorrhea, multicystic ovaries, hypoplastic uteri, elevated estradiol and gonadotropin levels, as well as delayed bone age [65–68]. Only one 46,XX individual with a heterozygous *ESR2* mutation has been described. This person presented with absent breast development, a small uterus, hypoplastic labia majora, and absent ovaries [69]. Functional analyses, when performed, showed reduced but not absent receptor activity (Table 1). Therefore, no complete oestrogen insensitivity has been described in 46,XX individuals so far.

**Table 1:** Clinical and functional data on human ESR variants; *ERE: Estrogen response element

mutation	clinical data	functional data	ref
*ESR1*:c.1125G > T; p.Gln375His (homozygous)	primary amenorrhea, no breast development, small uterus with no clearly identifiable endometrial stripe, enlarged multicystic ovaries, elevated serum E2 levels, delayed bone age	ERE* reporter assays show significantly reduced ESR1 activity of mutated compared to wild-type ESR1 at lower doses of estradiol reaching wild-type activity at higher doses.	[67]
*ESR1*:c.1181G > A; p.Arg394His (homozygous)	primary amenorrhea, no breast development, small uterus and thin endometrium, enlarged multicystic ovaries, high serum E2 levels and elevated gonadotropin levels, delayed bone age	ERE reporter assays show significantly reduced ESR1 activity of mutated compared to wild-type ESR1 at lower doses of estradiol reaching wild-type activity at higher doses.	[65]
*ESR1*:c.1628T > C; p.Met543Thr (homozygous)	no breast development, rudimentary uterus, stenosis of the vaginal orifice, polycystic ovaries, elevated E2 and gonadotropin levels, delayed bone age	not reported	[66]
*ESR1*:c.1214 C > T, p.Ala405Val (homozygous)	no breast development, rudimentary uterus, enlarged ovaries with multiple cysts, right ovarian torsion, imperforate hymen, mild clitoronegaly, high oestrogen and gonadotropin levels, delayed bone age	not reported	[68]
*ESR2*:c.941 A > G, p.Lys314Arg (heterozygous)	primary amenorrhea, no breast development, eunuchoid habitus, small infantile uterus, no ovaries, hypoplastic labia majora, non-oestrogenized infantile vulva, delayed bone age	ERE reporter assays show significantly reduced ESR2 activity of mutated compared to wild-type ESR2 at all doses of estradiol tested but a much smaller yet still significant reduction of ESR2 activity when combining wild-type and mutant ESR2 (as seen in the patient). The authors conclude a dominant negative effect of the mutation.	[69]

Another view that aligns with the concept of female-passive sex development regards the regression of the Wolffian ducts. Current models explain the degeneration of the Wolffian ducts in the female with the lack of actively stabilizing androgens. Again, this goes back to Jost´s initial castration experiments showing that androgens are needed for the stabilization and development of the Wolffian ducts. The common accepted conclusion, that the lack of androgens is providing a full explanation of Wolffian duct regression might have limited further research for years. Only in 2017, a factor necessary for the regression of the Wolffian ducts was discovered in female mice. The authors showed that female mouse embryos lacking *Nr2f2* in the Wolffian duct mesenchyme retain their ducts independently of androgen action [70]. Subsequently, in humans, several mutations in *NR2F2* in individuals with 46,XX difference of sex development were described. The clinical features included no palpable gonads and virilization of the genitalia. In two cases 46,XX ovotesticular DSD was diagnosed and Wolffian remnants were detected [71, 72]. Since the regression of the Wolffian ducts was considered to occur in the absence of androgens, no genetic factors were considered to be relevant in individuals with 46,XX DSD and therefore a molecular diagnosis and potential treatment options were lacking for these individuals. This emphasizes how a delay in research due to unquestioned assumptions has a direct impact on female health.

**Fig. 3:**
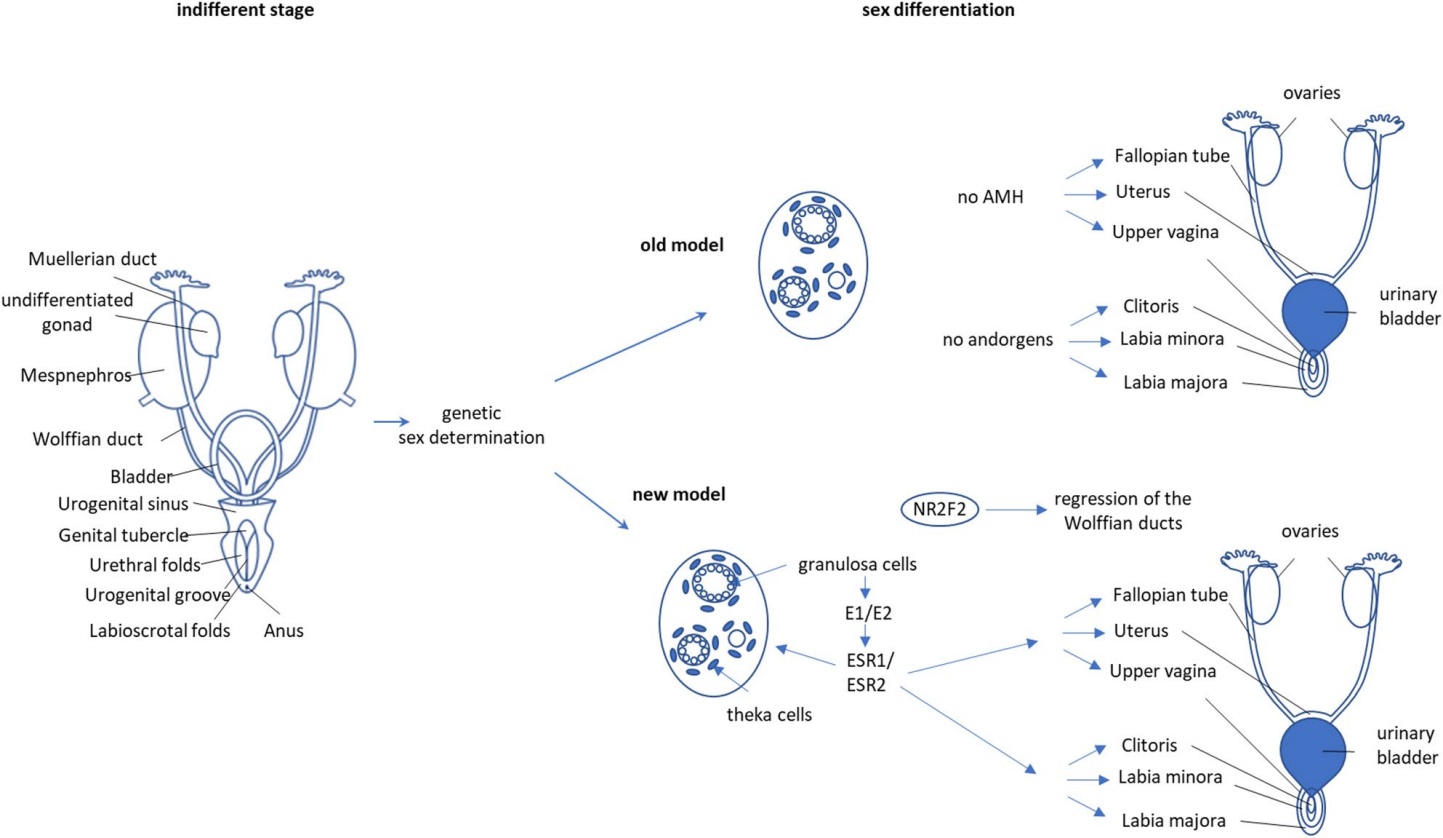
Updated model of female sex development

In conclusion, the above-mentioned findings directly challenge the prevailing scientific notion that addresses the development of female genitalia as arising solely from the absence of testicular hormones. While it is clear that excess of androgens counteracts female sex development and that physiologically low levels of androgens are necessary for female sex development, the absence of androgens is not sufficient to produce a female genital. In short, femaleness is not just the absence of maleness - it requires its own set of developmental instructions, particularly from oestrogen signalling (Fig. 3). Future research is needed to better understand the role of oestrogens in female sex development.

Why did the concept of absence become an accepted body of knowledge that went largely unchallenged for a long time? Although the perfect human model for fetal oestrogen action (complete oestrogen insensitivity) is still missing, this limitation cannot explain the lack of knowledge in female sex development research. One explanation could be that the research interest in studying passiveness or absence is not very appealing. There is no reason studying something, which is labeled “passive”, as it has no added value. Therefore, the central question that arises is whether sociocultural constructions of gender shape the ways in which female sex development is described and understood.

## Symbolic constructions of femininity in cultural contexts

### Absence and the epistemology of sex development

The idea that female sex development follows a passive path - occuring merely in the absence of male signals - was neither scientifically uncontested nor epistemically self-evident. As the preceding sections have shown, the interpretative routines associated with the concept of absence are not merely empirical linear developing theoretical models. In biomedical research, an understanding of developmental mechanisms is always constructed within technologically intensive and context-specific experimental settings. These artificial environments are shaped by infrastructural and methodological factors, as well as epistemic presuppositions, disciplinary research cultures, and model-theoretical choices, which actively co-construct the objects and dynamics of knowledge production [2, 7, 12].

As feminist science studies have demonstrated for years, cultural notions of masculinity and femininity have consistently influenced scientific inquiry, shaping the questions asked, the methods used, and the interpretations offered. These influences often obscured complexity, reinforced binary explanatory models and shaped research premises, such as the biological concept of the female sex development as a passive trait [6, 7, 9, 12, 13, 73–75].

This historical framing of research has been closely intertwined with broader cultural assumptions about femininity. In this context, ‘femininity’ refers to gendered cultural norms and socially constructed traits [76–78], while ‘femaleness’ designates biological sex categories that are themselves culturally interpreted rather than purely natural [13]. The biological presence/absence model perpetuates a narrative in which activity, agency, and value are associated with masculinity, while femininity is depicted as passive, default, or absent [79, 80]. This seems to render femininity unworthy of being the subject of research [13].

A substantial body of feminist science studies has already documented how processes coded as ‘passive’ or associated with femininity have historically received less analytical attention or have been conceptualized as secondary or default option. For instance, Emily Martin’s analysis of reproductive biology reveals that ovulation has traditionally been described using metaphors of passivity, in contrast to the highly active portrayal of sperm [80]. Similarly, Londa Schiebinger [81] demonstrates that, scientific debates in the 18th and 19th centuries, disproportionately focused on male anatomy and physiology, while research on the female bodies - particularly on menstruation, ovulation, and embryonic development - was often marginalized or considered derivative. Recognizing the conceptual labor involved in the notions of ‘absence’ enables to critically reflect on what is overlooked or undervalued.

A striking example of this phenomenon is the application of Alfred Jost’s theory of genital development to animal behavior studies [13]. When Jost states that “Masculine characteristics of the body have to be imposed in males […] against a basic feminine trend of the mammalian body” ([82] cited in [13]), it becomes evident that the concept of absence, which is rooted in cultural norms that devalue femininity, has also emerged in the production of biomedical knowledge. Fausto-Sterling demonstrates that, in the mid-20th-century, developmental biology conceptualized femaleness as a “natural starting template” and, consequently, as a form of bodily absence. In contrast, maleness was framed as requiring active, interventionist processes. This rhetorical asymmetry had methodological consequences as female development was considered the default and hormone-independent mechanism, researchers saw little need to investigate its mechanisms in detail [13]. To this day, the scientific literature documents far more information on the factors influencing testicular tissue activation than on ovarian development. This imbalance reflects a persistent epistemic divide, which Crasnow and Intemann describe as either “willful or accidental negligence” or the “absence of appropriate conceptual resources” [83]. 

Moreover, non-knowledge is not merely a lack of information [84], but rather the active production of constructed non-knowledge. This process must be understood as both a scientific and social practice. Consequently, feminist theorists have extensively examined the concept of femininity as absence, emphasizing the significance of non-knowledge and its intricate interplay with the concepts of sex and gender [85]. The pejorative connotation of ‘absence’ as a deficiency is problematic in two respects: First, absence is more than a mere term; it is a concept that shapes and guide’s research, and causes the production of non-knowledge, which is not marked as such. This phenomenon of an active production of non-knowledge is described in social science using the concept of epistemic ignorance in der Klammer [86–89]. Second, despite empirical evidence to the contrary, absence remains an established and, above all, frequently reiterated concept till today.

### Feminist theory of femininity as absence

In feminist theory, ‘absence’ is a central concept, particularly in analyses of cultural assumptions about sex and gender differences. Simone de Beauvoir argued that femininity is not defined through its own attributes, but rather through what it is not - not male, not rational and not thinking. Consequently, femininity appears as a negation or absence of the masculine [90]. Her question “What is a woman?” provides the starting point for a critique of the symbolic and material production of women as non-subjects in patriarchal societies. In this framework, femininity functions as a projection surface for cultural, patriarchal ideas, being passive and symbolically constructed as ‘other’.

Luce Irigaray furthers this critique by analyzing the symbolic erasure of the feminine in Western metaphysics. Drawing on Lacan’s assertion that “woman does not exist” [91], she exposes how patriarchal discourse consistently frames femininity in terms of what is lacking in language and meaning. In this sense, femininity is constructed culturally as an absence and embedded within dominant structures of thought.

However, both Irigaray and Beauvoir offer more than just a critique. Their central question they ask is: How can femininity be reimagined, beyond the symbolic framework of absence? How can the feminine be understood as an autonomous, symbolic presence rather than a void? In Irigaray’s work in particular, we see attempts to create new symbolic orders in which femininity is no longer invisible, but positively conceivable [92]. Since then, there has been an extensive discussion about the meanings and consequences of cultural concepts of femininity and their impact on scientific bodies of knowledge and practices. This is a debate that continues to this day, particularly with regard to the lived experiences of women.

Consequently, such gaps in knowledge can affect research outcomes and manifest as concrete health risks for women. For example, there is insufficient evidence on sex-specific pharmacological responses, and women bear a disproportionate burden of cardiovascular morbidity and mortality due to gendered diagnostic as well as research biases that obscure female symptomatology and constrain evidence-based therapeutic strategies [93, 94]. This issue has been criticized by feminist scholars since the 1970 s and continues to be discussed in the broader fields of social research and public debate [95, 96–98].

### Toward reflexive and inclusive scientific practice

In recent years several initiatives by organizations such as the NIMH, NIH, and the EU [99, 100, 101] have importantly raised awareness of androcentric and gender bias in biomedical research. This has been achieved by developing new methodological instruments, such as considering “sex as biological variable” (SABV) when developing research hypotheses or analyzing scientific data. While these concepts are valuable institutional steps, their full potential depends on the embedding of sex as a complex, relational, and contextual variable alongside comprehensive analysis. This requires new forms of interdisciplinary dialogue to overcome the gap between the ‚two cultures‘ of sex and gender [19, 20, 102]. Without integrating more nuanced and dynamic concepts of sex and gender, these policies risk perpetuating biological determinism rather than resolving the underlying conceptual issues [93, 102].

Fausto-Sterling once argued, “we can do a different - and, I believe, better - job of envisioning human sexuality without falling into the nature/nurture abyss.” [13]. Thus, rethinking absence as a state of lack, passivity, or silence becomes essential for developing more reflexive, inclusive, and transformative science. This requires a critical and reflexive engagement with scientific concepts to improve terminology, methods, and theoretical frameworks.

In this spirit, Longino emphasizes a fundamental condition of scientific practice: creating spaces for negotiation where critique can be voiced within a pluralistic, discursive framework. Such spaces enable engagement with diverse perspectives and methods [103, 104]. This is a call to improve our ways of knowing, by addressing androcentric bias in empirical data, and by encouraging interdisciplinary dialogue to improve our understanding of how knowledge is produced and why certain forms of knowledge emerge, while others remain out of reach.

## Conclusions

The history of the 'absence-theory' of female sex development illustrates how scientific knowledge has been deeply entangled with cultural assumptions and epistemic biases. While 20th-century experimental models established androgens as active drivers of male differentiation, they simultaneously framed female development as a passive default process defined only by the absence of testicular hormones. This concept limited research for decades, leaving the mechanisms of ovarian and oestrogen signalling understudied, which in turn had direct consequences for female health. A critical review of the literature shows that female genital development is an active process shaped by genes, oestrogens and ovarian signalling, and it underscores the need for more reflexive research approaches. To advance beyond established scientific models, it is crucial to acknowledge how cultural contexts shape scientific frameworks and to foster an interdisciplinary approach that integrates biology, history, and feminist epistemology in order to produce more accurate, nuanced, and equitable accounts of sex development knowledge. Moving beyond established sex-based models also requires institutional commitment to more reflective research practices. These practices can realize their full potential by treating sex and gender as complex, dynamic, and interrelated context-dependent variables, thereby avoiding the reinforcement of biologically determinist assumptions.

## Supplementary Information

Supplementary Material 1

## Data Availability

No datasets were generated or analysed during the current study.
