# Supplementary material for: The persisting presence of absence in female sex development: a critical interdisciplinary reflection
Source: Biol Sex Differ. 2026 Mar 25;17:71. doi: 10.1186/s13293-026-00848-2 (PMC13063871; doi:10.1186/s13293-026-00848-2)
Supplement: Supplementary file 1 — Supplementary Material 1 [file 13293_2026_848_MOESM1_ESM.docx]

| **References** | **Examples of using the absence-concept in human female sex differentiation** |
| --- | --- |
| Ray, R. & Racine, C. (2025): Sexual Differentiation. In: *Endotext*. MDText.com, Inc. (Bookshelf ID: NBK279001), PMID: 25905232. | “Subsequently, internal and external genitalia will follow the male pathway in the presence of androgens and anti-Müllerian hormone (AMH), or the female pathway in their absence.” |
| Aatsha, P. A., Arbor, T. C., & Krishan, K. (2023): Embryology, Sexual Development. In: *StatPearls*. StatPearls Publishing (Bookshelf ID: NBK557601), PMID: 32491533. | “The absence of testosterone in females, and some males, leads to regression of the Wolffian duct during sexual differentiation.”  “Female external genital development is regulated by the absence of androgens and the presence of maternal estrogens.” |
| Lumen learning: Biology for Majors II, Module 16: The Reproductive System, Introduction to human reproductive Anatomy, <https://courses.lumenlearning.com/wm-biology2/chapter/introduction-to-human-reproductive-anatomy/> (last access: 2026, Jan 20). | “Testosterone causes the undeveloped tissues to differentiate into male sexual organs. When testosterone is absent, the tissues develop into female sexual tissues.” |
| AMBOSS SE. (2025): Geschlechtsentwicklung – Zusammenfassung (Sektion: Klinische Praxis), <https://next.amboss.com/de/article/Qo0ubS> (last access 2026, Jan 10). | „Die weitere geschlechtliche Differenzierung verläuft beim Mann hormonabhängig, während die weiblichen Geschlechtsorgane ohne hormonelle Einflüsse entstehen.”  English translation: Further sexual differentiation in males proceeds in a hormone-dependent manner, whereas the female reproductive organs develop without hormonal influence. |
| Chapter 628: Disorders of sex development – sex differentiation. In: Kliegman, R. M., Blum, N. J., Tasker, R. C., Wilson, K. M., St. Geme, J. W., III, Schuh, A. M., Mack, C. L., & Deardorff, M. A. (eds.) (2024): *Nelson textbook of pediatrics*. 22nd ed., 2-vol. set. Elsevier. | “Antimüllerian hormone (AMH) causes the müllerian (paramesonephric) ducts to regress; in its absence they persist as the uterus, fallopian tubes, cervix, and upper vagina.” p. 3503  “In the XX-fetus, with normal short and long arms of the X-chromosome, the bipotential gonad develops into an ovary by about the 10^th^ to 11^th^ week. This occurs only in the absence of SRY, testosterone, and AMH […].” p. 3503  “A female external phenotype develops in the absence of fetal gonads. However, the male phenotype development requires androgen production and action. Estrogen is unnecessary for normal prenatal sexual differentiation […].” p. 3503 |
| Chapter 8.12: Disorders of sex development. In: Hamdy, F. C. & Eardly, I. (eds.) (2017): *Oxford Textbook of surgical urology*. Oxford University Press. | “Unlike in male, differentiation of the internal and external genitalia in the female occurs as a passive process which is not dependent upon exposure to sex hormones at different stages in the differentiation pathway.” p. 1015 |
| Carlson, B. M. (2018): *Human Embryology and Developmental Biology*. 6th ed. Elsevier. | “In the absence of specific testicular influences or the ability to respond to them, a female phenotype results.” p. 372 |
| Chapter 75: Development and congenital anomalies of the urogenital system. In: Brennan, P. A., Standring, S., & Wiseman, S. (eds.) (2019): *Gray’s surgical anatomy*. 1st ed. Elsevier, pp. 560–569. | “Absence of a Y-chromosome initiates a female development pathway. The coelomic epithelial cells cluster around each primordial germ cell and become granulosa (follicular) cells, which form primordial ovarian follicles.” pp. 560–561 |
| Torchia, M. G. & Persaud, T. V. N. (2024): *The Developing Human: Clinically Oriented Embryology*. 12th ed. Elsevier. | “The absence of a Y chromosome results in the formation of an ovary.” p. 244  “Primary female sexual differentiation does not depend on hormones, it occurs even if the ovaries are absent […].” p. 244 |
| Chapter 12: Urogenitalsystem. In: Persaud, T. V. N. & Torchia, M. G. (eds.) (2026): *Embryologie. Entwicklungsstadien, Frühentwicklung, Organogenese, Klinik*. 7. Aufl. Urban & Fischer Verlag/Elsevier, pp. 277–320. | „Die primäre Differenzierung zum weiblichen Geschlecht hangt allerdings möglicherweise gar nicht von besonderen weiblichen Faktoren oder Hormonen ab; sie findet zum großen Teil sogar statt, sobald das Y-Chromosom fehlt, sodass man bei der weiblichen Entwicklung im englischen Sprachgebrauch auch vom ‚default type‘ (also dem ‚voreingestellten‘ Typ) spricht. Der Typus der jeweiligen Gonade legt dann die weitere Geschlechtsentwicklung (in den Genitalgängen und im äußeren Genitale) fest. Der fetale Hoden bestimmt durch Testosteron-Produktion den männlichen Phänotyp und sorgt mit der Produktion des sog. Anti-Müller-Hormons (AMH) für die Unterdrückung der (‚voreingestellten’) weiblichen Entwicklung […].“ p. 298  English translation: However, the primary female sex differentiation may not depend on specific female factors or hormones; to a large extent, it occurs as soon as the Y chromosome is missing, which is why female development is referred to in English as the ‘default type’. The type of gonad then determines further sexual development (in the genital ducts and external genitalia). The foetal testis determines the male phenotype through testosterone production and, through the production of the so-called anti-Müllerian hormone (AMH), suppresses the (‘default’) female development […].  „Bei weiblichen Embryonen bilden sich die Wolff-Gänge zurück, weil das Testosteron fehlt; die Müller-Gänge entwickeln sich dagegen weiter, weil das Anti-Müller-Hormone (AMH/MIS) fehlt. Die weibliche Geschlechtsentwicklung setzt sich also auch ohne Ovarien oder Geschlechtshormone fort, dabei bilden die Müller-Gänge den überwiegenden Teil der weiblichen Genitalwege.“ p. 304  Englisch translation: In female embryos, the Wolffian ducts regress because testosterone is absent; by contrast, the Müllerian ducts continue to develop because anti-Müllerian hormone (AMH/MIS) is absent. Female sexual development therefore proceeds even in the absence of ovaries or sex hormones, with the Müllerian ducts forming the majority of the female genital tract. |
| Hurtado, A., Mota-Gómez, I., Lao, M., Real, F. M., Jedamzick, J., Burgos, M., et al. (2024): Complete male-to-female sex reversal in XY mice lacking the miR-17~92 cluster. In: *Nature communications* 15(1). PMID: 38714644, DOI: 10.1038/s41467-024-47658-x. | “The Y-linked gene, SRY, is the trigger that breaks this balance: its expression in the pre-supporting cell lineage of the undifferentiated gonads from XY individuals induces testis differentiation, whereas its absence in XX gonads results in ovarian development.” p. 1  “In the embryonic testis, Sertoli and Leydig cells produce hormones (AMH and testosterone, respectively) that masculinise the embryo. In the absence of these hormones, XX embryos develop as  phenotypic females.” p. 1 |
| Reyes, A. P., León, N. Y., Frost, E. R., & Harley, V. R. (2023): Genetic control of typical and atypical sex development. In: *Nature reviews*. *Urology* 20(7), pp. 434–451. PMID: 37020056, DOI: 10.1038/s41585-023-00754-x. | “Sex development relies on the sex-specific action of gene networks to differentiate the bipotential gonads of the growing fetus into testis or ovaries, followed by the differentiation of internal and external genitalia  depending on the presence or absence of hormones.” p. 434  “Finally, sexual differentiation is dictated by the presence or absence of hormones, which differentiates the internal and external genitalia for males and females.” p. 435 |
| Luppino, G., Wasniewska, M., Coco, R., Pepe, G., Morabito, L. A., Li Pomi, A., Corica, D., & Aversa, T. (2024): Role of *NR5A1* Gene Mutations in Disorders of Sex Development: Molecular and Clinical Features. In: *Current Issues in Molecular Biology* 46(5), pp. 4519–4532. PMID: 38785542, DOI: 10.3390/cimb46050274. | “In contrast, the differentiation of female genitalia depends on the absence of a Y chromosome, AMH and testosterone.” p. 4521 |
| Lopez Dacal, J., Castro, S., Suco, S., Correa Brito, L., Grinspon, R. P., & Rey, R. A. (2024): Assessment of testicular function in boys and adolescents. In: *Clinical endocrinology* 101(5), pp. 455–465. PMID: 37814597, DOI: 10.1111/cen.14979. | “Androgens and AMH drive male genital differentiation; in their absence, female differentiation of the genitalia occurs.” p. 457, Fig. 2 |

**Supplement table 1**: Examples of the persistence of the absence-concept in current scientific literature as well as in currently used textbooks for university teaching and health professionals.
